# Effects of dietary replacement of soybean meal with dried distillers grains with solubles on the microbiota occupying different ecological niches in the rumen of growing Hu lambs

**DOI:** 10.1186/s40104-020-00499-2

**Published:** 2020-09-07

**Authors:** Junshi Shen, Zhipeng Li, Zhongtang Yu, Weiyun Zhu

**Affiliations:** 1 Laboratory of Gastrointestinal Microbiology, Jiangsu Key Laboratory of Gastrointestinal Nutrition and Animal Health, College of Animal Science and Technology, Nanjing Agricultural University, Nanjing, 210095 China; 2 National Center for International Research on Animal Gut Nutrition, Nanjing Agricultural University, Nanjing, 210095 China; 3 Department of Special Animal Nutrition and Feed Science, Institute of Special Animal and Plant Sciences, Chinese Academy of Agricultural Sciences, Changchun, 130112 China; 4 Department of Animal Sciences, The Ohio State University, Columbus, OH 43210 USA

**Keywords:** Distillers dried grains with solubles, Growing lamb, Microbiota, Ruminal ecological niche, Soybean meal

## Abstract

**Background:**

Diet has a profound impact on the rumen microbiota, and the impact can vary among the different rumen ecological niches (REN). This study investigated the effects of dietary replacement of soybean meal (SBM) with dried distillers grains with solubles (DDGS) on the rumen microbiota occupying different REN of growing Hu lambs. After a 9-week feeding trial, 6 lambs from each dietary treatment (SBM vs. DDGS-based diets) were slaughtered for sample collection. The microbiota of the rumen solid, liquid, and epithelium fractions was examined using amplicon sequencing analysis of bacterial 16S rRNA gene, functional prediction, and qPCR.

**Results:**

No interaction of dietary protein source (PS) and REN were detected for virtually all the measurements made in this study. The DDGS substitution resulted in very limited influence on bacterial community structure. However, the metabolic pathways predicted from 16S rRNA gene sequences varied greatly between SBM- and DDGS-based diets. The populations of rumen total bacteria, fungi, sulfate-reducing bacteria (SRB), and methanogens were not influenced by DDGS substitution, but the population of protozoa was reduced. The bacterial communities in rumen solid (RS) and liquid (RL) were similar in taxonomic composition but were different in relative abundance of some taxa. In contrast, the bacterial composition and relative abundance of rumen epithelium (RE) were greatly distinct from those of the RS and the RL. In alignment with the bacterial relative abundance, the metabolic pathways predicted from 16S rRNA genes also varied greatly among the different REN. The populations of total bacteria, protozoa, and methanogens attached to the RE were smaller than those in the RS and RL, and the fungal population on the rumen epithelium was smaller than that in the RS but similar to that in the RL. On the contrary, the SRB population on the RE was greater than that in the RS and RL.

**Conclusions:**

Substitution of SBM with DDGS had greater impact to the protozoa than to the other microbes, and the microbial community structure and functions at different REN are distinct and niche-adapted.

## Background

Rumen microbiota determines animal health and production performance, and a better understanding of this complex and diverse microbiota, especially the relationship between the structure and function under different dietary conditions can lead to innovative interventions to improve animal productivity [1]. Dietary changes can shift rumen microbiota with respect to its composition and structure and fermentation patterns [2, 3]. Dried distillers grains with solubles (DDGS) is an abundant byproduct of ethanol production from grains (primarily corn), which are high in crude protein (CP), ether extract (EE) and energy content [4, 5], and low in cost [6]. It has been widely used as a substitute for corn and soybean meal (SBM) in ruminant production [7, 8]. Several studies have evaluated the impact of feeding DDGS on rumen bacterial community in steers [9–11] and dairy cattle [12, 13]. However, the influences of DDGS addition on rumen microbial community structure and function in growing lambs remain poorly understood. In our previous study using growing lambs, substituting DDGS for SBM in an isonitrogenous diet increased dietary EE, neutral detergent fiber (NDF) and acid detergent fiber (ADF) contents, and significantly altered rumen fermentation parameters such as volatile fatty acid (VFA) and ammonia concentrations [5].

Within the rumen ecosystem, the environments create three different ruminal ecological niches (REN), namely the solid, liquid, and epithelium [14, 15]. The three REN have different community structure [15–18] and rumen function [17, 19]. Previous studies have compared the bacterial community occupying the three REN using polymerase chain reaction-denaturing gradient gel electrophoresis (PCR-DGGE) [17, 20–22], cloning [16, 21] or high-throughput sequencing [15, 18, 19, 23–25]. To our knowledge, most of these studies using dairy cattle or steers as experimental animals, and only one study, which used PCR-DGGR [17], investigated the bacterial community occupying the different REN of growing lambs. Recently, we also evaluated the changes of the bacterial communities at the three different REN of growing Hu lambs in response to dietary urea supplementation [26]. However, most of the studies focused only on the community structure and function of rumen bacteria, without considering the eukaryotes [27]. This study, by integrating real-time quantitative polymerase chain reaction (qPCR), high-throughput sequencing, and functional prediction, investigated the effect of dietary replacement of SBM with DDGS on community structure and function of the microbiota at the three different REN of growing Hu lambs.

## Methods

### Animals, diets and experimental design

The experimental design, diets, and management have been reported previously [5]. Briefly, The DDGS replaced all the SBM and a portion of the ground corn in the diets. The feeding trial was conducted for 10 weeks, with the first 1 week for adaptation followed by 9 weeks of dietary treatment. At the end of week 9, six lambs each were randomly selected only from the SBM-Control and the DDGS-Control groups and slaughtered for sample collection.

### Sample collection

On days 6 and 7 of week 9, the six lambs each selected from the SBM-Control and DDGS-Control were slaughtered at 4–6 h after morning feeding. The whole rumen contents of each Hu lamb were first homogenized, mixed, and then strained through four layers of cheesecloth to separate the rumen liquid (RL) and the rumen solid (RS) fractions. Approximately 30 mL each of liquid and the solid fraction was collected into a sterilized tube and immediately stored in liquid nitrogen. To collect the rumen epithelial (RE) samples, the rumen walls were rinsed with cold sterile saline solution three times after the removal of the rumen contents. Epithelial tissue samples from an approximately 8 cm^2^ area of the rumen epithelium were scraped using a sterilized glass slide and stored in liquid nitrogen immediately after collection. The RS, RL, and RE samples were stored at − 80 °C until further analysis.

### DNA extraction

Metagenomic DNA of the rumen solid, liquid, and epithelium samples was extracted using the bead-beating and phenol-chloroform extraction method as previously described [28]. The quality of the DNA extracts was visually checked using electrophoresis on 1.2% agarose gel (w/v) containing Goldview™ (SaiBaiSheng, Shanghai, China), and the DNA concentration of each sample was determined using a Nanodrop 2000 (Thermo Fisher Scientific, Inc., Madison, USA). The DNA samples were stored at − 20 °C until analyses.

### Illumina sequencing of 16S rRNA gene amplicons and data analysis

The V3-V4 hypervariable regions of the 16S rRNA gene were amplified using primers 338F (5^′^-ACTCCTACGGGAGGCAGCA-3^′^) and 806R (5^′^-GGACTACHVGGGTWTCTAAT-3^′^). Unique barcodes were added to the 5^′^ end of both primers for multiplexing. PCR products were verified on agarose gel (2%, w/v), and the expected bands were each extracted and purified using the AxyPrepDNA Gel Extraction Kit (Axygen Biosciences, CA, USA). The concentrations of the purified DNA amplicons were each quantified using a QuantiFluor® dsDNA kit (Promega, Madison, WI, USA). Amplicons from different samples were mixed in equal ratio and sequenced using the 2 × 300 paired-end kit on an Illumina MiSeq platform. The raw sequence reads were deposited into the NCBI Sequence Read Archive (SRA) database under the accession number PRJNA565493.

Raw fastq files were de-multiplexed, quality-filtered, and analyzed using QIIME 1.9.1 [29]. Operational taxonomic units (OTUs) were *de novo* clustered with a 97% sequence similarity cutoff using UPARSE (version 7.1 http://drive5.com/uparse/), and possible chimeric sequences were identified and removed using UCHIME [30]. The most abundant sequence within each OTU was selected as the ‘representative sequence’ and was taxonomically classified based on the SILVA database (version 128) [31]. A PH Lane mask supplied by QIIME was used to remove the hypervariable regions from the aligned sequences. FASTTREE [32] was used to create a phylogenetic tree of the representative sequences for each sample. Sequences identified as of chloroplasts or mitochondria were removed before further analysis. Alpha diversity measurements including observed OTUs, Chao1 richness estimate, and Shannon diversity index, as well as Good’s coverage, were calculated using QIIME 1.9.1 [29]. Principal coordinates analysis (PCoA) was performed based on weighted UniFrac distance and Bray-Curtis dissimilarity to reveal overall differences in prokaryotic communities among the rumen solid, liquid and epithelial fractions from SBM and DDGS groups (SBM-RS, SBM-RL, SBM-RE, DDGS-RS, DDGS-RL, and DDGS-RE). The functional profiles of the rumen microbiota from different REN of the growing Hu lambs were predicted from the 16S rRNA gene data using Tax4Fun [33]. The functional profiles were summarized at hierarchy level 2 of Kyoto Encyclopedia of Genes and Genomes (KEGG) pathways.

### Quantitative real-time PCR analysis

The PCR primers used for real-time qPCR of total bacteria [34], fungi [34], protozoa [35], methanogens [36], and SRB [37] are listed in Table S1. Real-time PCR was performed on a StepOnePlus system (Applied Biosystems, California, USA) using the SYBR Premix Ex Taq dye (Takara Bio Inc.). Copies of 16S rRNA gene (total bacteria), 18S rRNA gene (fungi and protozoa), methyl coenzyme-M reductase alpha subunit gene (*mcr*A, for methanogens), and dissimilatory sulfite reductase alpha subunit gene (*dsr*A, for SRB) in each sample was quantified in three technical replicate against respective standards, which were purified PCR products of known length and concentration. The absolute abundance of each microbial population was expressed as copies of the target gene/g of samples.

### Statistical analyses

Analysis of similarity (ANOSIM) was used to assess the statistical significance of the PCoA analysis of overall microbiota across the treatments. The real-time PCR data were log transformed to improve normality. Residual analysis was used to determine if a transformation of variables was needed. If needed, cubic root transformations were performed. All data (absolute abundance quantified by qPCR, alpha diversity measurements, relative abundances of microbial populations at the phylum and genus levels, and the relative abundance of level 2 KEGG pathways) were analyzed using the MIXED procedure of SAS version 9.4 (SAS Institute Inc., Cary, NC). The model included PS and REN as fixed effects, with block as random effects. Degrees of freedom were calculated using the Kenward-Roger option. The mean separation test was performed using the Tukey multiple range test. Differences were considered statistically significant at *P* ≤ 0.05.

## Results

### Effect on bacterial alpha diversity

Across all 36 samples, a total of 1,275,639 quality-checked 16S rRNA gene sequences were obtained. On average, each sample had at least 35,434 sequences. Greater than 99.3% depth coverage was achieved for all the treatments (Table 1). There were no interactions (*P* ≥ 0.06) between PS and REN for OTU numbers, Chao 1, or Shannon index. None of the alpha diversity measurements was affected (*P* ≥ 0.36) by dietary PS. The number of OTUs and Chao 1 richness estimate in the RS and the RL fractions were similar (*P* > 0.05), which were higher (*P* < 0.05) than in the RE fraction. In contrast, The Shannon index was higher (*P* > 0.05) in the RS than in the RL and the RE fraction.

**Table 1:** Effect of replacing soybean meal with dried distillers grains with solubles on the alpha diversity measurements of ruminal microbiota (at 3% dissimilarity level) of different ruminal ecological niches of growing Hu lambs

Item	Protein source (PS)			Ruminal ecological niche (REN)				*P*-value		
	SBM	DDGS	SEM	Solid	Liquid	Epithelial	SEM	PS	REN	PS × REN
Coverage, %	99.3	99.3	0.03	99.3	99.3	99.3	0.03	0.36	0.14	0.43
Observed OTUs	650.6	667.3	27.50	711.5^a^	694.3^a^	571.1^b^	31.02	0.57	< 0.01	0.33
Chao 1	820.1	837.1	26.66	870.6^a^	861.0^a^	754.2^b^	31.80	0.63	0.02	0.48
Shannon index	4.39	4.39	0.089	4.73^a^	4.34^b^	4.11^b^	0.109	0.98	< 0.01	0.06

^a-b^Means with different superscripts within a row differ (*P* < 0.05)

### Effect on overall bacteria

**Fig. 1:**
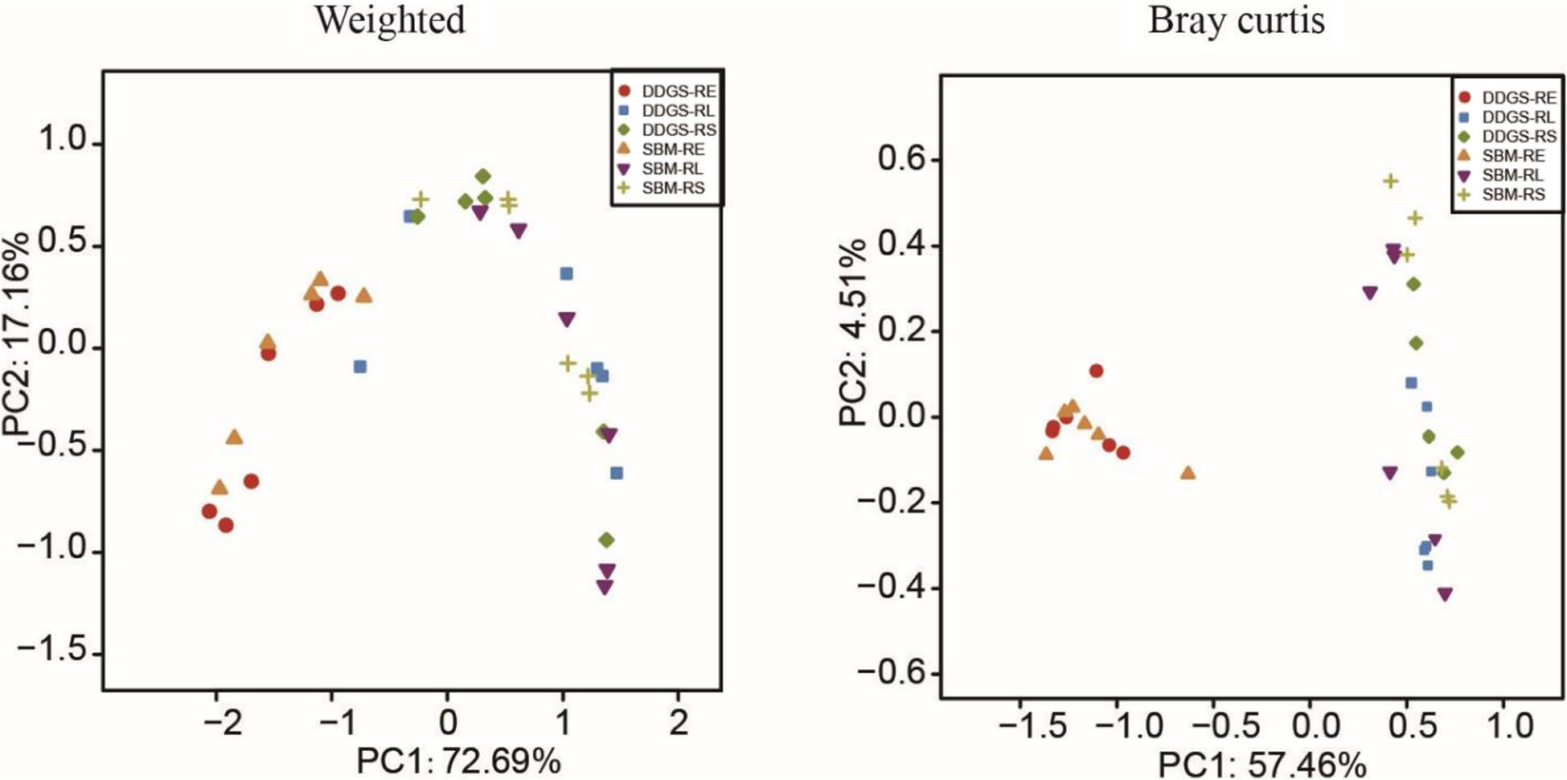
Principal coordinates analysis (PCoA) plots showing the comparison of the overall rumen bacteria in different ruminal ecological niches of growing Hu lambs. PCoA based on UniFrac distance (left) or Bray-Curtis dissimilarity (right). DDGS: dried distillers grains with solubles, SBM: soybean meal, RE: rumen epithelium, RS: rumen solid, RL: rumen liquid

The PCoA analysis, based on either Weighted UniFrac or Bray-Curtis dissimilarity, indicated a clear separation between the epithelium and the liquid or solid fraction, but no dietary PS effects were observed (Fig. 1). Moreover, the samples from the RS and the RL fractions clustered closely together, indicating very similar community structures. These findings were further supported by the ANOSIM based on the Bray-Curtis distance (*P* < 0.001; Figure S1).

### Effect on bacteria at phylum and genus levels

In total 19 bacterial phyla were identified among all the treatments, with Bacteroidetes, Firmicutes, Proteobacteria, Spirochaetes, Fibrobacteres, and Actinobacteria being the six most predominant phyla, each of which was represented by more than 0.5% of the total sequences in at least one treatment (Table 2). No interaction (*P* ≥ 0.59) of PS with REN was detected with respect to any of the bacterial phyla. None of the bacterial phyla was affected (*P* ≥ 0.11) by dietary PS, but all were greatly influenced by REN. The relative abundance of Bacteroidetes (52.15–55.01%) in the RS and the RL was higher (*P* < 0.01) than that in the RE fraction (27.41%). On the contrary, the relative abundance of Proteobacteria (0.64–0.93%) and Spirochaetes (0.76–2.45%) in the RS and the RL was lower (*P* < 0.01) than that in the RE fraction (18.06% and 6.23%). The relative abundance of Fibrobacteres was higher (1.16%) in the RS than in the RL and the RE fractions (0.12–0.30%). The relative abundance of Firmicutes (41.41–45.63%) and Actinobacteria (0.65–0.85%) were not influenced (*P* ≥ 0.55) by REN.

**Table 2:** Effect of replacing soybean meal with dried distillers grains with solubles on relative abundance of major ruminal bacterial phylum (each with a relative abundance ≥0.5% in at least one treatment) in different ruminal ecological niches of growing Hu lambs

Phylum	Protein source (PS)			Ruminal ecological niche (REN)				*P*-value		
	SBM	DDGS	SEM	Solid	Liquid	Epithelial	SEM	PS	REN	PS× REN
Bacteroidetes	47.80	41.92	2.779	52.15^a^	55.01^a^	27.41^b^	3.404	0.15	< 0.01	0.83
Firmicutes	40.65	45.21	3.034	41.75	41.41	45.63	3.716	0.30	0.68	0.78
Proteobacteria^1^	1.42 (6.05)	1.45 (7.04)	0.111	0.88^b^ (0.93)	0.84^b^ (0.64)	2.58^a^ (18.06)	0.120	0.68	< 0.01	0.47
Spirochaetes	3.10	3.20	0.859	2.45^b^	0.76^b^	6.23^a^	0.941	0.90	< 0.01	0.59
Fibrobacteres	0.49	0.56	0.214	1.16^a^	0.12^b^	0.30^b^	0.254	0.82	< 0.01	0.94
Actinobacteria	0.59	0.85	0.115	0.65	0.85	0.66	0.141	0.11	0.55	0.84

^a-b^Means with different superscripts within a row of REN differ (*P* < 0.05)

^1^Data were cubic root transformed to ensure normality of residuals. Value in parenthesis is the mean of untransformed data in each treatment

A total of 238 bacterial genera were identified among all the treatments, but only 49 of them were each represented by more than 0.5% of the total sequences in at least one treatment (Table 3) and they were regarded as the “major genera”. No interaction (*P* ≥ 0.13) of PS with REN was detected on any of the bacterial abundance at the genus level except for the genera *Pseudobutyrivibrio* and *Suttonella* (*P* < 0.01). The relative abundance of *Ruminococcaceae* UCG-005 was higher (*P* < 0.01) for the lambs receiving SBM than those fed DDGS, while the relative abundance of other major bacterial genera was not influenced (*P* ≥ 0.08) by PS. In contrast, 39 out of the 49 major genera were significantly different (*P* < 0.05) among the three REN. Compared with the RS and the RL fraction, the relative abundance of 17 genera (including *Butyrivibrio* 2, *Prevotellaceae* UCG-001, *Rikenellaceae* U29-B03, *Rikenellaceae* Blvii28, *Lachnospiraceae* UCG-008, *Defluviitaleaceae* UCG-011, *Eubacterium nodatum* group, *Erysipelotrichaceae* uncultured, *Lachnospiraceae* UCG-010, *Howardella*, *Halomonas*, *Desulfobulbus*, *Comamonas*, *Suttonella*, *Neisseriaceae* uncultured, *Campylobacter*, and *Treponema* 2) was higher (*P* < 0.01), while that of five genera (including *Prevotella* 1, *Prevotellaceae* UCG-003, *Christensenellaceae* R-7 group, *Ruminococcaceae* NK4A214 group, and *Lachnospiraceae* XPB1014 group) was lower (*P* < 0.01) in the RE fraction than in the RS and the RL fractions. Seven genera, including *Bacteroidales* S24-7 group_norank, *Succiniclasticum*, *Ruminococcus* 1, *Saccharofermentans*, *Eubacterium ruminantium* group, *Lachnospiraceae* probable genus 10, and *Fibrobacter*, had a greater relative abundance (*P* < 0.01) in the RS than in the RL and RE fractions. In contrast, the RL fraction had a higher (*P* < 0.01) relative abundance of *Bacteroidales* BS11 gut group_norank, *Bacteroidales* RF16 group_norank, *Ruminococcus* 2, *Eubacterium coprostanoligenes* group, and *Pseudobutyrivibrio*, but a lower (*P* < 0.01) relative abundance of *Ruminococcaceae* UCG-014, than the RS and the RE fractions.

**Table 3:** Effect of replacing soybean meal with dried distillers grains with solubles on relative abundance of major ruminal bacterial genera (each with a relative abundance ≥0.5% in at least one treatment) in different ruminal ecological niches of growing Hu lambs

Phylum	Genus/other	Protein source (PS)			Ruminal ecological niche (REN)				*P*-value		
		SBM	DDGS	SEM	Solid	Liquid	Epithelial	SEM	PS	REN	PS× REN
Bacteroidetes	*Prevotella* 1	19.19	19.50	2.779	25.01^a^	28.89^a^	3.94^b^	3.404	0.93	< 0.01	0.83
	*Rikenellaceae* RC9 gut group	7.09	5.76	0.589	6.59	7.13	5.57	0.721	0.12	0.31	0.39
	*Prevotellaceae* UCG-001	4.48	4.36	0.923	1.82^b^	1.23^b^	10.20^a^	1.131	0.93	< 0.01	0.93
	*Bacteroidales* S24-7 group	3.66	3.06	0.549	6.56^a^	2.42^b^	1.09^b^	0.672	0.45	< 0.01	0.25
	*Bacteroidales* BS11 gut group	2.81	2.61	0.655	2.18^b^	4.23^a^	1.72^b^	0.765	0.80	0.03	0.88
	*Prevotellaceae* Unclassified^1^	1.00 (3.79)	0.63 (0.36)	0.165	0.83 (1.54)	0.87 (3.97)	0.74 (0.72)	0.195	0.08	0.86	0.72
	*Prevotellaceae* UCG-003	1.73	1.13	0.347	1.61^a^	2.41^a^	0.28^b^	0.404	0.16	< 0.01	0.80
	*Bacteroidetes* Unclassified^1^	0.87 (1.24)	0.69 (0.48)	0.069	1.01^a^ (1.39)	0.89^a^ (1.07)	0.43^b^ (0.11)	0.085	0.08	< 0.01	0.96
	*Prevotellaceae* NK3B31 group	0.51	1.33	0.551	2.29	0.39	0.09	0.675	0.30	0.06	0.62
	*Bacteroidetes* VC2.1 Bac22	0.49	0.31	0.136	0.72^a^	0.38^ab^	0.10^b^	0.150	0.18	< 0.01	0.41
	*Bacteroidales* RF16 group	0.71	0.62	0.212	0.12^b^	1.64^a^	0.25^b^	0.212	0.66	< 0.01	0.97
	*Rikenellaceae* U29-B03^1^	0.53 (0.25)	0.60 (0.55)	0.062	0.46^b^ (0.12)	0.38^b^ (0.08)	0.84^a^ (1.00)	0.077	0.43	< 0.01	0.33
	*Rikenellaceae* Blvii28^2^				ND	ND	1.402				
Firmicutes	*Christensenellaceae* R-7 group^1^	1.86 (7.47)	2.01 (8.18)	0.111	1.99^ab^ (8.25)	2.21^a^ (11.79)	1.59^b^ (3.43)	0.136	0.36	0.01	0.76
	*Butyrivibrio* 2^1^	1.39 (5.95)	1.24 (5.18)	0.146	0.76^b^ (0.57)	0.79^b^ (0.61)	2.40^a^ (15.51)	0.156	0.16	< 0.01	0.65
	*Ruminococcaceae* NK4A214 group	3.63	4.38	0.461	4.66^a^	5.95^a^	1.40^b^	0.564	0.25	< 0.01	0.39
	*Succiniclasticum*	1.99	1.39	0.270	2.60^a^	0.99^b^	1.48^b^	0.330	0.13	< 0.01	0.92
	*Ruminococcus* 1	0.92	0.94	0.201	2.04^a^	0.58^b^	0.17^b^	0.245	0.95	< 0.01	0.84
	*Lachnospiraceae* NK3A20 group	1.70	2.26	0.308	2.33	2.00	1.61	0.375	0.20	0.40	0.97
	*Saccharofermentans*	0.82	1.03	0.112	1.88^a^	0.75^b^	0.16^c^	0.137	0.18	< 0.01	0.38
	*Ruminococcus* 2	1.33	1.47	0.484	0.98^b^	3.11^a^	0.11^b^	0.559	0.80	< 0.01	0.76
	*Eubacterium coprostanoligenes* group	0.69	0.58	0.068	0.74^b^	1.08^a^	0.10^c^	0.082	0.25	< 0.01	0.13
	*Ruminococcaceae* UCG-014	0.91	1.08	0.112	1.10^a^	0.55^b^	1.33^a^	0.138	0.28	< 0.01	0.37
	*Eubacterium ruminantium* group	0.32	0.44	0.102	0.87^a^	0.19^b^	0.07^b^	0.113	0.24	< 0.01	0.48
	*Lachnospiraceae* XPB1014 group^1^	0.64 (0.60)	0.81 (1.08)	0.085	0.93^a^ (1.35)	0.86^a^ (1.10)	0.38^b^ (0.07)	0.104	0.18	< 0.01	0.60
	*Ruminococcaceae* UCG-005	0.70	0.23	0.078	0.36	0.41	0.63	0.096	< 0.01	0.11	0.95
	*Lachnospiraceae* probable genus 10	0.24	0.34	0.093	0.694^a^	0.135^b^	0.046^b^	0.113	0.47	< 0.01	0.81
	*Ruminococcaceae* UCG-010	0.46	0.41	0.063	0.58^a^	0.45^ab^	0.29^b^	0.078	0.60	0.048	0.19
	*Lachnospiraceae* uncultured	0.30	0.38	0.108	0.63^a^	0.32^ab^	0.07^b^	0.125	0.56	< 0.01	0.79
	Family XIII AD3011 group	0.51	0.50	0.052	0.51	0.42	0.58	0.063	0.86	0.23	0.98
	*Pseudobutyrivibrio*^3^	0.44	1.06	0.135	0.56^b^	1.62^a^	0.06^b^	0.165	< 0.01	< 0.01	< 0.01
	*Lachnospiraceae* Unclassified^1^	0.73 (0.45)	0.83 (0.81)	0.097	0.82 (0.70)	0.74 (0.63)	0.77 (0.56)	0.105	0.23	0.69	0.39
	*Lachnospiraceae* AC2044 group^1^	0.48 (0.18)	0.54 (0.55)	0.074 0.199	0.73^a^ (0.82)	0.44^ab^ (0.20)	0.37^b^ (0.07)	0.090	0.54	0.02	0.92
	*Anaerovibrio*	0.28	0.25	0.069	0.25^ab^	0.51^a^	0.03^b^	0.084	0.75	< 0.01	0.97
	*Anaerovorax*	0.26	0.45	0.102	0.27^ab^	0.13^b^	0.65^a^	0.102	0.20	0.02	0.24
	*Lachnospiraceae* UCG-008^1^	0.85 (1.34)	0.73 (0.65)	0.049	0.58^b^ (0.21)	0.50^b^ (0.13)	1.29^a^ (2.65)	0.060	0.11	< 0.01	0.32
	*Defluviitaleaceae* UCG-011	0.30	0.25	0.089	0.16^b^	0.07^b^	0.59^a^	0.109	0.69	< 0.01	0.97
	*Eubacterium* nodatum group^1^	0.80 (1.10)	0.88 (1.45)	0.037	0.57^b^ (0.20)	0.46^b^ (0.11)	1.49^a^ (3.52)	0.045	0.16	< 0.01	0.69
	*Erysipelotrichaceae* uncultured	0.38	0.42	0.068	0.20^b^	0.11^b^	0.89^a^	0.077	0.61	< 0.01	0.98
	*Lachnospiraceae* UCG-010^1^	0.37 (0.21)	0.36 (0.16)	0.034	0.15^b^ (0.009)	0.16^b^ (0.008)	0.78^a^ (0.545)	0.042	0.86	< 0.01	0.32
	*Howardella*^1^	0.52 (0.43)	0.58 (0.61)	0.031	0.14^c^ (0.004)	0.40^b^ (0.071)	1.11^a^ (1.49)	0.038	0.14	< 0.01	0.67
Proteobacteria	*Halomonas*^1^	0.63 (0.56)	0.54 (0.50)	0.038	0.26^b^ (0.03)	0.40^b^ (0.07)	1.10^a^ (1.49)	0.046	0.10	< 0.01	0.26
	*Desulfobulbus*^1^	0.64 (1.47)	0.74 (2.03)	0.056	0.13^b^ (0.004)	0.28^b^ (0.031)	1.66^a^ (5.22)	0.069	0.24	< 0.01	0.39
	*Comamonas*^1^	0.35 (0.44)	0.36 (0.45)	0.057	0.03^b^ (0.001)	0.07^b^ (0.002)	0.96^a^ (1.34)	0.098	0.91	< 0.01	0.97
	*Suttonella*^1,3^	0.42 (0.31)	0.28 (0.08)	0.028	0.06^c^ (0.002)	0.22^b^ (0.016)	0.76^a^ (0.562)	0.034	< 0.01	< 0.01	< 0.01
	*Neisseriaceae* uncultured^2^				ND	ND	2.66				
	*Campylobacter*^2^				ND	ND	5.62				
Spirochaetes	*Treponema* 2	2.90	3.05	0.851	2.44^b^	0.74^b^	5.74^a^	0.932	0.84	< 0.01	0.51
Fibrobacteres	*Fibrobacter*	0.49	0.55	0.213	1.15^a^	0.11^b^	0.30^b^	0.235	0.83	< 0.01	0.94

^a-c^Means with different superscripts within a row of REN differ (*P* < 0.05)

^1^Data were cubic root transformed to ensure normality of residuals. Value in parenthesis is the mean of untransformed data in each treatment

^2^Genus/other can not be detected the rumen solid and liquid fraction, and only the difference of relative abundance of bacterial genera from rumen epithelium (RE) between SBM and DDGS (SBM-RE and DDGS-RE) was analyzed with SAS. Relative abundance (least square mean) of Blvii28, *Neisseriaceae* uncultured, and *Campylobacter* (%) for SBM-RE and DDGS-RE were 1.52 and 1.28 (*P* = 0.68); 2.52 and 2.80 (*P* = 0.88); 4.62 and 6.62 (*P* = 0.50), respectively

^3^Relative abundance (least square mean) of *Pseudobutyrivibrio* (%) was 0.52^b^, 0.74^b^, 0.07^b^, 0.61^b^, 2.52^a^ and 0.06^b^ for SBM-RS, SBM-RL, SBM-RE, DDGS-RS, DDGS-RL, and DDGS-RE, respectively, and that of *Suttonella* was 0.001^b^, 0.023^b^, 0.899^a^, 0.002^b^, 0.009^b^, and 0.225^b^ for SBM-RS, SBM-RL, SBM-RE, DDGS-RS, DDGS-RL, and DDGS-RE, respectively. DDGS: dried distillers grains with solubles, SBM: soybean meal, RE: rumen epithelium, RS: rumen solid, RL: rumen liquid

### Effect on inferred functional pathways

The top 20 predominant level 2 metabolic pathways were shown in Table 4. No interaction (*P* ≥ 0.10) of PS with REN was detected for any of the pathways. However, the relative abundance of the predominant pathways was altered differently by dietary PS or REN. Among the pathways, 9 out of 20 were significantly influenced by PS. Compared with the SBM-based diets, the DDGS-based diets resulted in higher (*P* ≤ 0.02) relative abundance of the functions related to amino acid metabolism, lipid metabolism, and metabolism of terpenoids and polyketides, but lower (*P* ≤ 0.04) relative abundance of the functions related to nucleotide metabolism, replication and repair, translation, cell motility, folding, sorting and degradation, infectious disease (bacterial), and cell growth and death. In contrast, 16 out of the predominant 20 pathways were significantly different (*P* < 0.05) among the three REN. Compared with the RS and RL, the RE fraction had greater (*P* < 0.01) predominance of the pathways related to amino acid metabolism, lipid metabolism, xenobiotics biodegradation and metabolism, and metabolism of terpenoids and polyketides, but lower (*P* < 0.01) predominance of the function related to membrane transport, signal transduction, and cell motility. The pathways related to replication and repair, biosynthesis of other secondary metabolites, and endocrine system were higher (*P* ≤ 0.02) in the RS than in the RL and RE fractions. In contrast, a higher (*P* < 0.01) relative abundance of the pathways related to the metabolism of cofactors and vitamins, and a lower (*P* < 0.01) relative abundance of the pathway related to the metabolism of other amino acids were observed in the RL than in the RS or the RE fractions.

**Table 4:** Effect of replacing soybean meal with dried distillers grains with solubles on relative abundance of predominant predicted gene pathways in the different ruminal ecological niches of growing Hu lambs

Metabolism	Protein source (PS)			Ruminal ecological niche (REN)				*P*-value		
	SBM	DDGS	SEM	Solid	Liquid	Epithelial	SEM	PS	REN	PS × REN
Carbohydrate metabolism	10.68	10.83	0.058	10.79	10.63	10.85	0.070	0.08	0.096	0.74
Amino acid metabolism	6.78	7.02	0.066	6.72^b^	6.84^b^	7.14^a^	0.080	0.02	< 0.01	0.99
Energy metabolism	4.48	4.45	0.029	4.47	4.52	4.41	0.036	0.51	0.12	0.81
Nucleotide metabolism	4.74	4.57	0.051	4.79^a^	4.48^b^	4.69^ab^	0.063	0.02	< 0.01	0.86
Replication and repair	4.31	4.09	0.056	4.42^a^	4.07^b^	4.11^b^	0.069	< 0.01	< 0.01	0.96
Metabolism of cofactors and vitamins	3.38	3.28	0.036	3.25^b^	3.52^a^	3.22^b^	0.044	0.07	< 0.01	0.66
Membrane transport	2.71	2.63	0.050	2.81^a^	2.75^a^	2.46^b^	0.062	0.23	< 0.01	0.10
Signal transduction	2.49	2.36	0.056	2.51^a^	2.63^a^	2.13^b^	0.068	0.11	< 0.01	0.18
Translation	2.68	2.52	0.051	2.73^a^	2.49^b^	2.58^ab^	0.061	0.03	0.02	0.82
Cell motility	1.85	1.62	0.070	1.81^a^	2.11^a^	1.29^b^	0.085	0.03	< 0.01	0.40
Lipid metabolism	1.62	1.74	0.032	1.61^b^	1.56^b^	1.86^a^	0.039	< 0.01	< 0.01	0.75
Folding, sorting and degradation	1.56	1.50	0.014	1.58^a^	1.53^ab^	1.49^b^	0.018	< 0.01	< 0.01	0.97
Metabolism of other amino acids	1.36	1.39	0.019	1.40^a^	1.31^b^	1.41^a^	0.023	0.32	< 0.01	0.67
Glycan biosynthesis and metabolism	1.21	1.20	0.021	1.17	1.22	1.21	0.026	0.73	0.27	0.94
Biosynthesis of other secondary metabolites	1.08	1.07	0.008	1.11^a^	1.04^b^	1.07^b^	0.010	0.24	< 0.01	0.87
Xenobiotics biodegradation and metabolism	1.02	1.14	0.033	1.06^b^	0.90^c^	1.28^a^	0.040	0.01	< 0.01	0.95
Metabolism of terpenoids and polyketides	0.87	0.93	0.025	0.85^b^	0.84^b^	1.01^a^	0.027	< 0.01	< 0.01	0.21
Infectious diseases: Bacterial	1.56	1.50	0.014	1.58^a^	1.53^ab^	1.49^b^	0.014	< 0.01	< 0.01	0.97
Endocrine system	0.85	0.82	0.017	0.88^a^	0.81^b^	0.81^b^	0.021	0.17	0.02	0.24
Cell growth and death	0.64	0.61	0.009	0.63	0.63	0.61	0.011	0.04	0.25	0.31

^a-b^Means with different superscripts within a row differ (*P* < 0.05)

### Effect on total bacteria, sulfate-reducing bacteria, methanogens, fungi, and protozoa

No interaction (*P* ≥ 0.16) between PS and REN was detected with respect to the absolute abundance of total bacteria, SRB, methanogens, fungi, or protozoa (Table 5). The DDGS-fed lambs had a smaller (*P* = 0.04) population of protozoa than those fed SBM, but the population of total bacteria, SRB, fungi, and methanogens were similar (*P* ≥ 0.41). The populations of total bacteria and methanogens were lower (*P* < 0.05) in the RL fraction than in the RS fraction, but higher (*P* < 0.05) than in the RE fraction. The RS had a larger (*P* < 0.05) fungal population than the RL and the RE fractions, but no difference (*P* > 0.05) in the fungal population was noted between the RL and the RE fractions. Compared with the RS and the RL fractions, the RE fraction had a larger (*P* < 0.05) SRB population but a smaller (*P* < 0.05) protozoal population. However, no difference (*P* > 0.05) was observed in the populations SRB or protozoa between the RS and the RL fractions.

**Table 5:** Effect of replacing soybean meal with dried distillers grains with solubles on the absolute abundance total bacteria, sulfur-reducing bacteria, fungi, protozoa, and methanogens (log_10_ copies of the target genes/g sample) in different ruminal ecological niches of growing Hu lambs

Item	Protein source (PS)			Ruminal ecological niche (REN)				*P*-value		
	SBM	DDGS	SEM	Solid	Liquid	Epithelial	SEM	PS	REN	PS × REN
Total bacteria	12.32	12.31	0.066	12.81^a^	12.31^b^	11.83^c^	0.073	0.76	< 0.01	0.44
Sulfur-reducing bacteria	8.50	8.62	0.138	8.24^b^	8.03^b^	9.42^a^	0.154	0.41	< 0.01	0.89
Fungi	9.61	9.55	0.104	11.52^a^	8.65^b^	8.56^b^	0.128	0.69	< 0.01	0.26
Protozoa	8.93	8.52	0.156	9.46^a^	9.16^a^	7.55^b^	0.183	0.04	< 0.01	0.93
Methanogen	10.01	10.03	0.058	10.41^a^	10.15^b^	9.50^c^	0.071	0.79	< 0.01	0.16

^a-c^Means with different superscripts within a row differ (*P* < 0.05)

## Discussion

### Effects of dietary protein source on rumen microbiota structure and function

Diet is one of the main factors influencing the structure and function of rumen microbiota [2, 3]. In our previous feeding trial, replacing SBM in an isonitrogenous lamb diet with 20% DDGS increased dietary EE, NDF and ADF contents [5]. Therefore, we expected shifts of rumen bacterial community and structure. However, high throughput sequencing results (Tables 1, 2 and 3) revealed very limited influence of dietary PS on the bacterial community structure. These results are similar to a previous study that reported no or little difference in microbiota diversity and relative abundance of most bacteria in the rumen of crossbred steers fed 19.5% DDGS replacing corn bran [11] or dairy cows fed 20% DDGS replacing SBM [13]. In contrast, Callaway et al. [9] and Ramirez-Ramirez et al. [12] found drastic changes in rumen bacterial community structure when DDGS were added at 30% and 50% of the diet [dry matter (DM)]. The disparity in bacterial community structure among different studies may be due to differences in DDGS feeding level and the chemical composition of the diets [13]. Additionally, the difference of ruminant species, duration of experimental period, sequencing and data analysis methods may also contribute to the discrepancy in the bacterial community among studies.

Although the bacterial community structure in the rumen did not differ much between the two different protein sources, the metabolic pathways predicted from the 16S rRNA gene sequences varied greatly (Table 4). These results demonstrated that the substitution of SBM with DDGS had a greater impact to the rumen microbial function than to the bacterial composition. It is also suggested that small changes in the rumen bacterial community may lead to greater variations in metabolic pathways. Among the predicted metabolic pathways, the increase of lipid metabolism pathway in the DDGS treatment group might be related to the higher EE content in the diet. The changes in other metabolic pathways, such as amino acid metabolism, metabolism of terpenoids and polyketides, nucleotide metabolism, replication and repair, translation, cell motility, might also be related to changes in dietary composition, but the specific mechanism is still unclear. The application of multiple meta-omics, such as metagenomics, metatranscriptomics, metaproteomics, and metabolomics [27] in future studies may help further explain the effect of different protein sources on rumen metabolism.

Feed digestion and fermentation are the concerted functions of a variety of microorganisms including bacteria, fungi, protozoa, archaea and phages [27]. Therefore, the change of rumen fermentation parameters found in our previous feeding trial [5] might be closely associated with shifts of members of the rumen microbiota besides bacteria. This study showed that the populations of rumen total bacteria, fungi, SRB, and methanogens were not influenced by the dietary protein sources, but the population of protozoa was reduced by the DDGS substitution for SBM (Table 5). Protozoa can account for up to 50% of the rumen biomass and play an important role in the degradation of dietary fiber and protein [38]. Moreover, protozoa are also known to increase ammonia production [39]. In a previous meta-analysis, it has been reported that the elimination of protozoa from the rumen significantly decreased VFA and ammonia concentration [40]. Therefore, the decreased protozoal population might have primarily contributed to the reduced VFA and ammonia concentration in the DDGS group [5]. These findings indicate that, in addition to bacteria, the structure and function of other microbes such as protozoa, fungi, methanogens, and phages should also be studied in nutritional research.

### Effects of ruminal ecological niche on microbiota structure and function

Consistent with previous studies using PCR-DGGE [17] or high-throughput sequencing [15, 18, 26], RS and RL shared more bacteria when compared to RE. In addition, the predicted functions and the qPCR results of the microbial groups further demonstrated that there were great differences in the structure and function of the microbiota occupying different REN. This corroborates the niche partitioning of the rumen microbiota.

#### Differences in microbiota between rumen solid and liquid fractions

The Shannon diversity index in the RS is higher than in the RL (Table 1), which agrees with the findings of previous studies [15, 18]. The bacterial richness and taxonomic composition in the RS and RL were similar, suggesting continuous exchange between these two fractions [15]. However, some taxa had different relative abundance in these two niches, a finding consistent with other reports [15, 18, 26] and likely reflecting specialized niches related to digestion of soluble components vs. dietary fiber [23].

Rumen bacteria found in the RS fraction are mainly responsible for the initial and secondary degradation of feed and play an important role in fiber digestion, while the bacteria in the RL fraction are mainly involved in the fermentation of soluble nutrients and metabolic end products of feed digestion [15, 41]. *Ruminococcus* and *Fibrobacter* are the two main known fibrolytic bacterial genera in the rumen [42]. We indeed found a higher predominance of *Ruminococcus* 1 and *Fibrobacter* spp. in the RS than in the RL fractions of the growing lambs (Table 3). Some other genera, such as *Bacteroidales* S24-7 group, *Succiniclasticum*, *Saccharofermentans*, *Eubacterium ruminantium* group, and *Lachnospiraceae* probable genus 10, were also more predominant in the RS than in the RL, which indicates that these bacteria may play an important role in the initial and secondary degradation of feed. In contrast, some other bacterial genera, such as *Bacteroidales* BS11 gut group, *Bacteroidales* RF16 group, *Ruminococcus* 2, *Eubacterium coprostanoligenes* group, and *Pseudobytyrivibrio* showed the opposite trend, which indicates that these bacteria probably are mainly involved in the catabolism of soluble nutrients. Moreover, the results of functional analysis further demonstrated that there were great differences in multiple bacterial metabolic pathways between the RS and the RL.

It should be noted that bacteria with similar relative abundance in the RS and the RL fraction may differ in their absolute abundance. In the present study, the population of total bacteria in the RS is much higher than that in the RL (Table 5), indicating that the population of bacteria might be greater in the RS than in the RL. In addition to bacteria, other rumen microorganisms such as protozoa, fungi and methanogens also play an important role in feed digestion [27]. However, recent studies mainly focused on the diversity and function of rumen bacteria, ignoring the structure and function of other microbial microbes [27]. The rumen fungi can account for up to 20% of the microbial biomass and play an important role in ruminal fiber degradation [43]. Methanogens are the main hydrogen utilizing microorganisms in the rumen, and hydrogen produced by hydrogen-producing bacteria such as cellulolytic bacteria and fungi are used by methanogens to reduce CO_2_ to methane [44]. Therefore, it is not surprising that the population of fungi and methanogens is higher in the RS than in the RL. In contrast, the similar population of protozoa between the RS and the RL in the rumen is probably due to the ability of protozoa to freely attach to and dissociate from feed particles [41]. Generally, the population of protozoa is more abundant than fungi in rumen [45]. In the present study, similar results was also found in the RL fraction. However, the population of fungi in RS fraction is much higher than protozoa. Therefore, the disparity with previous reports in the population of rumen fungi and protozoa may be due to differences in REN.

#### Differences in microbiota between epithelial and the solid or the liquid fractions

In line with previous studies [15, 18, 26], the RE bacterial diversity was lower than that in the RS or the RL fraction (Table 2), and the bacterial composition was greatly distinct from that of the RS or the RL (Table 3). At the phylum level, contrary to the finding in the RS or the RL, Firmicutes was the first while Bacteroidetes the second largest phyla in the RE microbiota. It is worth noting that Proteobacteria was the third largest phylum (with a relative abundance of 18.06%) of the RE microbiota, which is consistent with previous studies on dairy cattle [19, 24]. Members of Proteobacteria are mostly facultative anaerobes [46]. Therefore, the higher predominance of Proteobacteria on RE could be explained by the trace amounts of oxygen diffused through the rumen tissue [47]. Interestingly, all the dominant bacterial genera classified to Proteobacteria in the epithelial microbiota, such as *Campylobacter*, *Desulfobulbus*, *Neisseriaceae* uncultured, *Comamonas*, and *Halomonas* were barely detected in the RS or the RL. In addition to bacteria of Proteobacteria, some bacterial genera assigned to other phyla were predominant on the RE (e.g., *Prevotellaceae* UCG-001, *Butyrivibrio* 2, and *Treponema* 2), and again, they are virtually undetected or only detected at much lower relative abundance in the RS and or the RL. These results support the notion that the distinctive epithelial bacteria assigned to different phyla may have additional functions other than feed digestion [21].

Corroborating the finding of previous studies [26, 48, 49], *Desulfobulbus*, one genus of SRB [50], was the predominant SRB genus (5.22%) in RE microbiota (Table 3). In line with the high-throughput sequencing result, the qPCR results also demonstrated that SRB were more predominant on RE than in the RS or the RL fraction. Previous studies have shown that sulfides produced by SRB can disrupt the gut epithelial tissues and induce DNA damage, and adversely affect host health [51, 52]. However, it was found that the relative abundance of *Butyrivibrio* 2 (15.51%) on RE was about 3 times higher than that of *Desulfobulbus*. Members of *Butyrivibrio* (e.g., *Butyrivibrio fibrisolvens*) are important butyrate producing bacteria ubiquitous in the rumen [53]. *In vitro* cell culture experiments have shown that butyrate could regulate colonic proliferation and treat ulcerative colitis caused by sulfide [54]. Therefore, it is also reasonable to believe that butyrate, a metabolite of *Butyrivibrio* spp., may help repair the damage to the RE caused by the sulfide produced by SRB (e.g., *Desulfobulbus* spp.). Based on this, we speculate that there is a self-regulating mechanism between the epithelial microbes and the host that helps the homeostasis of RE function.

Rumen epithelial microbiota play an important role in digesting and recycling the keratinized distal cells of the epithelium [14]. In the present study, the enhanced pathway of amino acid metabolism and lipid metabolism in the RE microbiota compared with the RS and the RL microbiota (Table 4) are probably related with the special nutrient composition of the keratinized distal cells of the epithelium. One recent metatranscriptomic analysis of RE microbiota showed that many metabolic genes encoding enzymes involved in N metabolism such as glutamate dehydrogenase, glutamine synthase and glutamate synthase were highly expressed, which demonstrated the importance of RE bacteria in N metabolism [55]. Similarly, Mao et al. [19] also found enhanced amino acid metabolism in the RE microbiota compared with the microbiota of rumen content. In the present study, the qPCR results confirmed the existence of fungi, protozoa and methanogens in the epithelial microbiota. However, Mann et al. [55] detected fungi and methanogens but no protozoa on the RE in their metatranscriptomic analysis. The inconsistent results might be explained by the more sensitive detection by qPCR than by RNA-seq.

## Conclusions

This study, combing high-throughput sequencing, functional prediction, and qPCR, for the first time, revealed the difference of structure and function of rumen microbiota occupying different REN of growing Hu lambs in response to alterations in dietary PS. The results of the present study indicate that substitution of SBM with DDGS had greater impact to protozoa than to other microbes, and the microbiota structure and function in different REN are specific and niche-adapted. In order to have a better understanding of the complex rumen ecosystem, in addition to bacteria, the structure and function of other microbes such as protozoa, fungi, and methanogens should also be studied.

## Supplementary information

**Additional file 1 : Table S1**. Primers used for real-time PCR quantification of rumen target organisms. **Figure S1.** Box plots showing within-group similarity and between-group dissimilarity of rumen microbiota based on Bray-Curtis dissimilarity in different ruminal ecological niches of growing Hu lambs in response to alterations in dietary protein sources. The different letters denote significant differences (Kruskal-Wallis tests, FDR-adjusted q < 0.05). DDGS: dried distillers grains with solubles, SBM: soybean meal, RE: rumen epithelium, RS: rumen solid, RL: rumen liquid.

## Data Availability

All data generated or analyzed during this study are included in this published article [and its supplementary information files].

## Abbreviations

ADF: Acid detergent fiber
ANOSIM: Analysis of similarity for multivariate
CP: Crude protein
DDGS: Dried distillers grains with solubles
DE: Digestible energy
DM: Dry matter
DNA: Deoxyribonucleic acid
*dsr*A: Dissimilatory sulfite reductase alpha subunit gene
EE: Ether extract
KEGG: Kyoto Encyclopedia of Genes and Genomes
*mcr*A: Methyl coenzyme-M reductase alpha subunit gene
N: Nitrogen
NDF: Neutral detergent fiber
OTUs: Operational taxonomic units
PCoA: Principal coordinates analysis
PCR-DGGE: Polymerase chain reaction-Denaturing gradient gel electrophoresis
PS: Protein source
qPCR: Real-time quantitative polymerase chain reaction
RE: Rumen epithelium
REN: Ruminal ecological niche
RL: Rumen liquid
RS: Rumen solid
SBM: Soybean meal
SRB: Sulfur-reducing bacteria
TMR: Total mixed ration
VFA: Volatile fatty acid
