# Supplementary material for: Effects of dietary replacement of soybean meal with dried distillers grains with solubles on the microbiota occupying different ecological niches in the rumen of growing Hu lambs
Source: J Anim Sci Biotechnol. 2020 Sep 7;11:93. doi: 10.1186/s40104-020-00499-2 (PMC7487462; doi:10.1186/s40104-020-00499-2)
Supplement: Supplementary file 1 — Additional file 1 : Table S1. Primers used for real-time PCR quantification of rumen target organisms. Figure S1. Box plots showing within-group similarity and between-group dissimilarity of rumen microbiota based on Bray-Curtis dissimilarity in different ruminal ecological niches of growing Hu lambs in response to alterations in dietary protein sources. The different letters denote significant differences (Kruskal-Wallis tests, FDR-adjusted q < 0.05). DDGS: dried distillers grains with solubles, SBM: soybean meal, RE: rumen epithelium, RS: rumen solid, RL: rumen liquid. [file 40104_2020_499_MOESM1_ESM.docx]

**Supplementary files**

Table S1. Primers used for real-time PCR quantification of rumen target organisms

| Target organisms | Primers sequences (5'→3') | Annealing T, °C | Amplicon length, bp | References |
| --- | --- | --- | --- | --- |
| Total bacteria | F: CGGCAACGAGCGCAACCC | 60 | 161 | Denman and McSweeney [34] |
|  | R: CCATTGTAGCACGTGTGTAGCC |  |  |  |
| Fungi | F: GAGGAAGTAAAAGTCGTAACAAGGTTTC | 60 | 120 | Denman and McSweeney [34] |
|  | R: CAAATTCACAAAGGGTAGGATGATT |  |  |  |
| Protozoa | F: GCTTTCCGWTGGTAGTGTATT | 54 | 223 | Sylvester et al. [35] |
|  | R: CTTGCCCTCYAATCGTWCT |  |  |  |
| Methanogens (*mcr*A gene) | F: TTCGGTGGATCDCARAGRGC | 60 | 190 | Denman et al. [36] |
|  | R: GBARGTCGWAWCCGTAGAATCC |  |  |  |
| Sulfate-reducing bacteria (*dsr*A) | F: CCAACATGCACGGYTCCA | 60 | 270 | Devkota et al. [37] |
|  | R: CGTCGAACTTGAACTTGAACTTGTAGG |  |  |  |

**
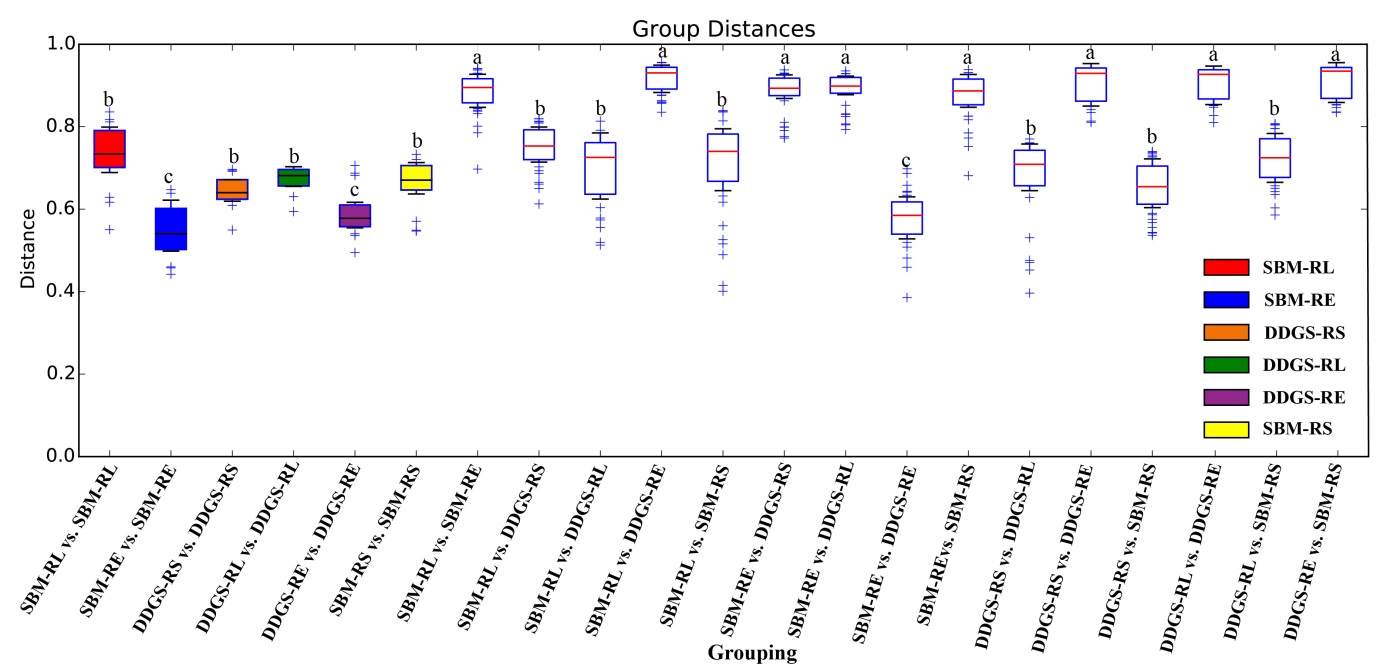
**

**Figure S1.** Box plots showing within-group similarity and between-group dissimilarity of rumen microbiota based on Bray-Curtis dissimilarity in different ruminal ecological niches of growing Hu lambs in response to alterations in dietary protein sources. The different letters denote significant differences (Kruskal-Wallis tests, FDR-adjusted q<0.05). DDGS: dried distillers grains with solubles, SBM: soybean meal, RE: rumen epithelium, RS: rumen solid, RL: rumen liquid.
